# Associations Between Blood Eosinophil Surface Proteins and Clinical Traits in Severe Asthma and Chronic Rhinosinusitis With Nasal Polyposis 

**DOI:** 10.1111/all.70001

**Published:** 2025-08-02

**Authors:** Emeline Delaunay, Stephane Esnault, Arnaud Dendooven, Thomas Stoup, Thibaut Vanderhaegen, Louison Jan, Laurine Cadart, Cecile Chenivesse, Geoffrey Mortuaire, Guillaume Lefèvre

**Affiliations:** ^1^ Univ. Lille, INSERM, CHU Lille, U1286—INFINITE—Institute for Translational Research in Inflammation Lille France; ^2^ CHU Lille, Institut D'immunologie, Médecine Interne et Immunologie Clinique Lille France; ^3^ Division of Allergy, Pulmonary and Critical Care Medicine, Department of Medicine University of Wisconsin‐Madison School of Medicine and Public Health Madison Wisconsin USA; ^4^ Univ. Lille, CHU Lille, Service de Pneumologie et Immuno‐Allergologie, U1286 Inserm INFINITE, Univ. Lille, Institute for Translational Research in Inflammation Lille France; ^5^ Univ. Lille, CHU Lille, Service D'otorhinolaryngologie, U1286 Inserm INFINITE, Univ. Lille, Institute for Translational Research in Inflammation Lille France; ^6^ CHU Lille, Service de Biostatistique Lille France; ^7^ Univ. Lille, CHU Lille, CNRS, Inserm, Institut Pasteur Lille, U1019‐UMR9017‐CIIL‐Centre D'infection et D'immunité de Lille Lille France; ^8^ CRISALIS (Clinical Research Initiative in Severe Asthma: A Lever for Innovation & Science) Toulouse France

**Keywords:** CRSwNP, disease severity, eosinophils, severe asthma, surface protein expression

To the Editor,

Severe asthma and chronic rhinosinusitis with nasal polyposis (CRSwNP) are airway diseases mostly caused by type 2 (T2) inflammation, in which eosinophils play an important role [1]. Severe asthma is characterised by persistent symptoms despite high doses of corticosteroids, which can lead to long‐term side effects [2]. Consequently, biologics have been developed, including those targeting eosinophils through interleukin‐5 (IL‐5) or its receptor. However, these therapies do not uniformly suppress eosinophil activity [3], suggesting heterogeneity among eosinophil populations and differential sensitivity to treatments. In atopic dermatitis, another T2 inflammatory disorder, we have previously demonstrated that blood eosinophils display unique surface markers compared to healthy individuals [4]. Therefore, this study aimed to determine whether circulating eosinophils in patients with severe asthma or CRSwNP display altered surface marker expression in comparison to healthy controls. Furthermore, we assessed whether specific surface markers correlate with diseases clinical features.

The description and characteristics of the participants included in the study are presented in Table 1 and the Document S1. The levels of surface markers were examined by flow cytometry as described in the Document S1. Among the surface markers, CD123 and CD125 are critical receptors for interleukin‐3 (IL‐3) and IL‐5 involved in eosinophil differentiation, migration and survival. We also analysed Siglec‐8, a marker of mature eosinophils and mast cells that reduces eosinophil activity [5] and chemokine receptors, CCR3 and CRTH2, binding the eotaxins and prostaglandin D2, respectively. Additionally, we assessed adhesion molecules (CD44 and CD62‐L) and eosinophil activation markers (CD63, CD69, CD137, HLA‐DR).

**TABLE 1:** Characteristics of the participants.

	Healthy controls	Severe asthma	CRSwNP
*Demographics*			
Number of participants (Nb of part.)	19	24	16
Sex (% female)^a^	53	79	37
Age (years)^b^	38 ± 3.6 [38; 25; 50]	50 ± 3.6 [53; 40; 60]	55 ± 5.3 [51; 45; 61]
Blood eosinophils (per mm ^3^)^b^	117 ± 11.7 [100; 78; 153]	211 ± 37 [155; 84; 312]	305 ± 80.6 [203; 165; 296]
Nb of part. with eosinophilia (≥ 150/mm^3^)	6	12	12
Nb of part. with allergic rhinitis	NA	16	2
Nb of part. with atopic dermatitis	NA	6	0
*Clinical scores*			
Age at asthma onset		26 ± 4.6 [20; 11; 40]	
ACT		13 ± 0.7 [14; 12; 15]	
ACQ‐6		2.9 ± 0.2 [2,6; 2,3; 3,4]	
Exacerbations (last 12 months)		6.5 ± 1.5 [3,5; 2; 10,5]	
FEV_1_/FVC (%)		68 ± 3.1 [73; 56; 79]	
FeNO (ppb)		45 ± 9.2 [29; 14; 59]	
Dose ICS_Becl. Equiv (μg/day)		2437 ± 274 [2000; 1750; 3250]	
Total IgE (kIU/L)		294 ± 99 [95; 38; 302]	
VAS (%)			43 ± 2.8 [43; 36; 52]
SNOT‐22			47 ± 6.4 [46; 29; 65]
CT Lund Mac Kay			18 ± 1.2 [19; 18; 22]
NPS			5.1 ± 0.4 [5; 4; 6]

*Note:* Mean ± standard error and [median; Q1; Q3].

Abbreviations: ACQ‐6, Asthma Control Questionnaire; ACT, Asthma Control Test; Becl. Equiv, Beclomethasone equivalent; CRSwNP, chronic rhinosinusitis with nasal polyps; CT Lund Mac Kay, CT scan score of sinus opacification; FeNO, fractional exhaled nitric oxide; FEV_1_/FVC, forced exhaled volume in 1 s/forced vital capacity; ICS, inhaled corticosteroid; Nb of part., number of participants; NPS, nasal polyp score; SNOT‐22, Sino‐Nasal Outcome Test; VAS, visual analogic scale.

^a^ Chi‐square < 0.05 between AS and CRSwNP.

^b^ Kruskal–Wallis *p* < 0.05 between HC and CRSwNP.

Among all analysed markers, only CD125 was significantly higher on eosinophils from patients with severe asthma and in patients with CRSwNP compared to healthy controls (Figure 1A and Data S1 Figure 1B). We then performed correlation analyses between surface marker levels and clinical features (Figure 1B,C). In severe asthma, higher levels of HLA‐DR, CD69, and CD44 correlated with poorer asthma control, as determined by the Asthma Control Test (ACT) or the Asthma Control Questionnaire (ACQ‐6). Furthermore, increased airflow limitation, assessed via the Forced Exhaled Volume in 1 s/forced vital capacity ratio (FEV_1_/FVC), was associated with increased CD123 on eosinophils. CD62‐L expression inversely correlates with age at disease onset and fractional exhaled nitric oxide (FeNO), the latter of which indicates greater T2 airway inflammation in patients with low CD62‐L (Figure 1B). However, unlike Mesnil et al. [6], we did not identify distinct eosinophil subsets based on CD62‐L and CD123 in either patients or controls. The number of exacerbations, total serum IgE levels and inhaled corticosteroid dose did not show significant correlations (Figure S2A). In CRSwNP, CCR3 level inversely correlated with nasal polyp score (NPS), whereas CD63 showed a positive correlation (Figure 1C). These significant associations are further illustrated in Figure S2B. We also performed correlation analyses between surface marker expressions (Table S1). However, due to the limited sample size, we did not pursue more complex multivariate clustering approaches at this stage.

**FIGURE 1:**
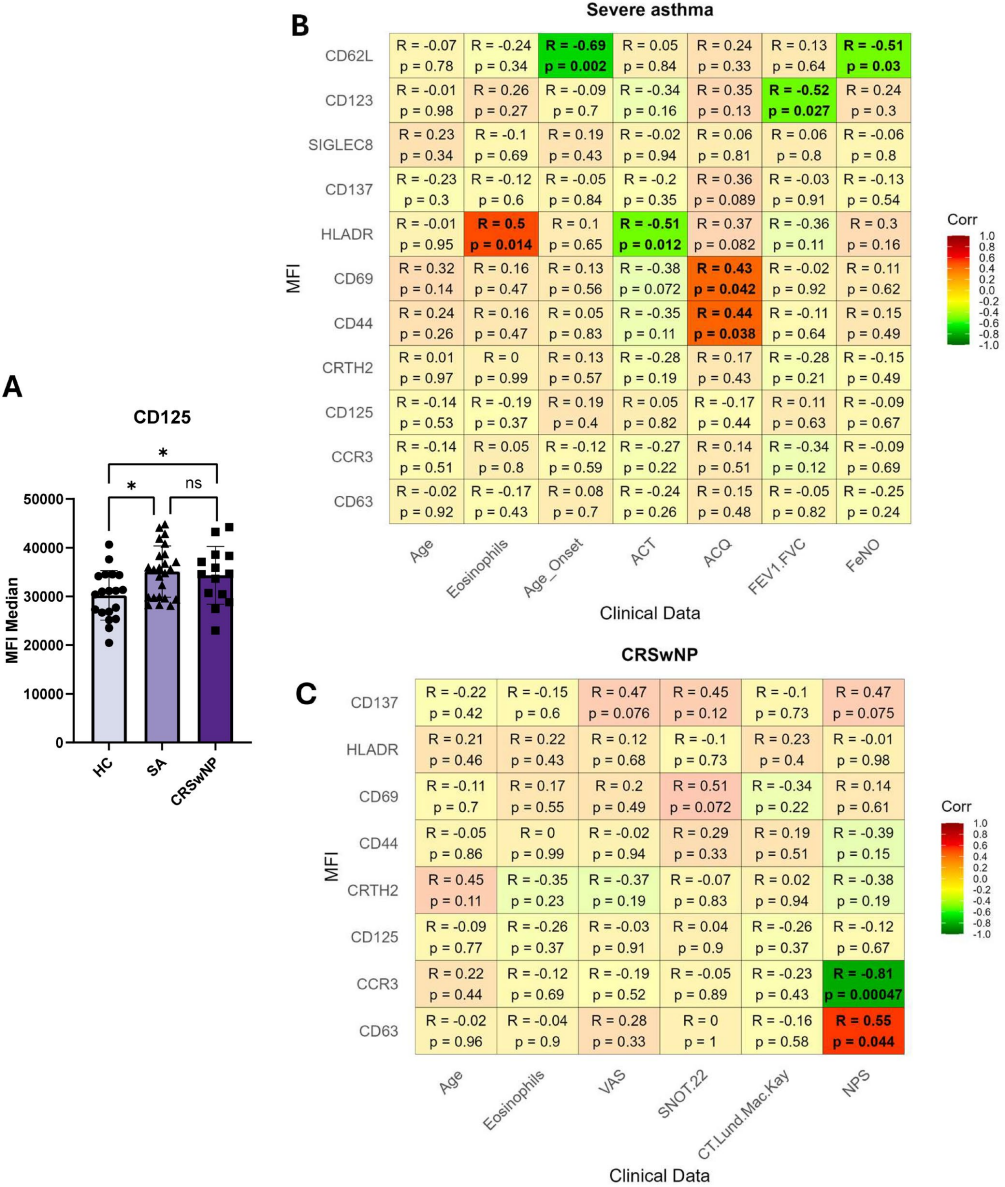
CD125 expression and marker correlations in severe asthma and CRSwNP. (A) Difference in expression of CD125 MFI (median fluorescent intensity) between the three groups: Healthy control (HC), severe asthma (SA) and CRSwNP (*n* = 19 HC; 24 SA; 14 CRSwNP). Adjustments were made for age and gender. Statistical analyses were performed with a ANCOVA test (*p < 0.05, ns = not significant). (B, C) Matrix of correlations between the expression of surface markers on blood eosinophils and the clinical score from the group (B) severe asthma and (C) CRSwNP. Correlations were calculated using Spearman's method. Statistical correlations (*p* < 0.05) are represented by brighter colours. CD62‐L, CD123 and Siglec‐8 were removed from the analysis for the CRSwNP group due to insufficient data. ACQ‐6, Asthma Control Questionnaire; ACT, Asthma Control Test; CRSwNP, chronic rhinosinusitis with nasal polyps; CT Lund MacKay, CT scan score of sinus opacification; eosinophils, eosinophils count in the blood; FeNO, fractioned exhaled nitric oxide; FEV_1_/FVC, forced exhaled volume in 1 s/forced vital capacity; NPS, nasal polyp score; *p*, *p*‐value of the test; *R*, Spearman factor; SNOT 22, Sino‐Nasal Outcome Test; VAS, visual analogic scale.

In conclusion, this is a study analysing human blood eosinophil surface markers in patients with severe asthma and CRSwNP. Variability among participants and limited sample size may have restricted the number of statistically significant outcomes. However, we observed a high surface expression of CD125 in severe asthma and CRSwNP compared to controls. This finding highlights the potential of using eosinophil phenotyping to identify patients who may respond to anti‐IL‐5 and IL‐5 receptor therapy despite a moderate eosinophil count. Furthermore, we identified specific eosinophil surface markers (i.e., HLA‐DR, CD69, CD44, CCR3) correlated with phenotypic and treatable traits (ACT, ACQ‐6, NPS), providing potential biomarkers of airway diseases. Further research is needed to confirm the utility of eosinophil surface protein profiling in predicting disease features and therapeutic response.

## Author Contributions

All authors have contributed significantly to the study.

## Conflicts of Interest

C. Chenivesse declares research grants from AstraZeneca, GSK, Santelys, and Novartis; personal fees from ALK‐Abello, AstraZeneca, Boehringer‐Ingelheim, Celtrion, Chiesi, Sanofi, and GSK; and congress support from AstraZeneca, Boehringer Ingelheim, Chiesi, Sanofi, and Novartis. G. Lefèvre received consulting fees, personal fees for advisory boards or meetings, and research funding from AstraZeneca and GSK. The rest of the authors declare that they have no relevant conflicts of interest.

## Supporting information


**Data S1:** all70001‐sup‐0001‐DataS1.zip.

## Data Availability

The data that support the findings of this study are available from the corresponding author upon reasonable request.

## References

[1] I. Striz , K. Golebski , Z. Strizova , et al., “New Insights Into the Pathophysiology and Therapeutic Targets of Asthma and Comorbid Chronic Rhinosinusitis With or Without Nasal Polyposis,” Clinical Science (London, England) 137 (2023): 727–753, 10.1042/CS20190281. PMC10195992 37199256

[2] V. Sood , L. Rogers , and S. Khurana , “Managing Corticosteroid‐Related Comorbidities in Severe Asthma,” Chest 160 (2021): 1614–1623, 10.1016/j.chest.2021.05.021. 34019864

[3] K. Eger , J. A. Kroes , A. Ten Brinke , and E. H. Bel , “Long‐Term Therapy Response to Anti‐IL‐5 Biologics in Severe Asthma‐A Real‐Life Evaluation,” Journal of Allergy and Clinical Immunology. In Practice 9 (2021): 1194–1200, 10.1016/j.jaip.2020.10.010. 33069885

[4] F. Dezoteux , P. Marcant , A. Dendooven , et al., “Enhanced Siglec‐8 and HLA‐DR and Reduced CRTH2 Surface Expression, Highlight a Distinct Phenotypic Signature of Circulating Eosinophils in Atopic Dermatitis,” Journal of Leukocyte Biology 117 (2025): qiaf023, 10.1093/jleuko/qiaf023. 39998842

[5] T. Kiwamoto , N. Kawasaki , J. C. Paulson , and B. S. Bochner , “Siglec‐8 as a Drugable Target to Treat Eosinophil and Mast Cell‐Associated Conditions,” Pharmacology & Therapeutics 135 (2012): 327–336, 10.1016/j.pharmthera.2012.06.005. 22749793 PMC3587973

[6] C. Mesnil , S. Raulier , G. Paulissen , et al., “Lung‐Resident Eosinophils Represent a Distinct Regulatory Eosinophil Subset,” Journal of Clinical Investigation 126 (2016): 3279–3295, 10.1172/JCI85664. 27548519 PMC5004964

