# Supplementary material for: Associations Between Blood Eosinophil Surface Proteins and Clinical Traits in Severe Asthma and Chronic Rhinosinusitis With Nasal Polyposis
Source: Allergy. 2025 Aug 2;80(12):3454–7. doi: 10.1111/all.70001 (PMC12666750; doi:10.1111/all.70001)
Supplement: Supplementary file 1 — Data S1: all70001‐sup‐0001‐DataS1.zip. [file ALL-80-3454-s001.zip › all70001-sup-0001-Supplementary informations.docx]

**Supplementary information:**

**Associations between blood eosinophil surface proteins and clinical traits in severe asthma and CRSwNP.**

Emeline Delaunay et al.

**Material and Methods:**

*Human subjects*

Conducted at the University Hospital of Lille, France, the study included 24 patients diagnosed with uncontrolled severe asthma according to ERS/ATS guidelines and uncontrolled symptoms (ACQ-6≥1.5), along with 16 patients presenting recalcitrant CRSwNP requiring sinus surgery. Medical failure in CRSwNP was defined as persistent disease despite three courses of oral corticosteroids and double dose of local corticosteroids over 12 months. In the clinical characteristics of CRSwNP, we included patient -reported outcomes: quality of life score (SNOT-22; Sinonasal outcome test 22), overall symptoms burden including nasal pruritus, anosmia, pain, obstruction, rhinorrhea, sneezing and bleeding (VAS; visual analogic scale). We measured mean endoscopic nasal polyp score (NPS) for both sides and Lund-MacKay sinus opacification score on preoperative CT scan. Asthma characteristics included age at onset, control scores (ACT and ACQ) and pulmonary function (FEV1/FVC for airflow limitation). Nineteen healthy individuals were also analyzed. Exclusion criteria for the three groups included active or former smokers with over 10 pack-years, autoimmune diseases, use of systemic glucocorticoids (or discontinued for at least four weeks), macrolides, theophylline, antileukotrienes in the previous four weeks, and biologics in the previous six months. Participants provided written consent, and the study was approved by the Institutional Review Board Sud-Est I (2019-A01457-50) and declared to the National Commission on Informatics and Liberty. The study complied with the Helsinki rules on biomedical research.

*Flow cytometry*

Blood was collected in EDTA (disodium salt of ethylenediaminetetraacetic acid) tubes for each participant, erythrocytes were lysed using TQ-Prep (Beckman-Coulter®) and washed 2 times with PBS. Eosinophils were analyzed by flow cytometry with a CytoFLEX S Flow Cytometer across three panels containing CD45-KO and CD16-PB (Beckman-Coulter®). The other markers studied are CD63-FITC (Beckman-Coulter®), CD193 (CCR3)-APC, CD294 (CRTH2)-PeVio770 (Miltenyi-Biotec®), CD125-PE (BD-Biosciences®), CD44-FITC, CD69-APC (Beckman-Coulter®), HLA-DR-PE (BD-Biosciences®), CD137-PEVio615, Siglec-8-PEVio770, CD123-PEVio615 (Miltenyi-Biotec®), CD62L-APC-A750 (Beckman-Coulter®). Eosinophils were gated using a Forward Scatter (FSC) versus Side Scatter (SSC) plot to identify granulocytes (FSC^hi^/SSC^hi^), in which eosinophils were CD45+/ CD16- (see gating strategy in **Supplementary Figure 1A**). Data were analyzed with Median Fluorescence Intensity using Kaluza 2.1 software (Beckman Coulter®) and graph were made using GraphPad Prism.

*Statistical analysis*

Comparison between the 3 groups (healthy subjects, severe asthma and CRSwNP) were performed by analysis of covariance (ANCOVA) after adjustment for age and sex. Markers for which the normality of the residuals was not verified, even after log-transformation, were analysed by a non-parametric ANCOVA. The significance level was set at 5%. Statistical analyses were performed using SAS software (SAS Institute version 9.4). Correlation matrices were obtained with R studio software.

**Legends:**

**Supplementary Figure 1: Flow cytometry analysis of blood eosinophils**. **A)** Gating strategy to identify eosinophils from blood. **B)** Surface protein expression (median fluorescence intensity [MFI]) in the 3 groups: healthy control (HC), severe asthma (SA) and CRSwNP. CCR3 (n= 19 HC; 24 SA; 14 CRSwNP). CRTH2 (n= 19 HC; 24 SA; 14 CRSwNP). CD62-L (n= 18 HC; 18 SA; 6 CRSwNP). CD123 (n= 19 HC; 20 SA; 7 CRSwNP). Siglec-8 (n= 19 HC; 20 SA; 7 CRSwNP). CD69 (n= 19 HC; 23 SA; 15 CRSwNP). CD44 (n= 19 HC; 23 SA; 15 CRSwNP). HLA-DR (n= 19 HC; 23 SA; 15 CRSwNP). CD63 (n= 19 HC; 24 SA; 14 CRSwNP). CD137 (n= 19 HC; 23 SA; 15 CRSwNP). Adjustments were made for age and gender. Statistical analyses were performed with an ANCOVA test; p = p value for the global comparison (*p < 0.05 is significant); ns = not significant).

**Supplementary Figure 2: Correlation between surface marker expression on blood eosinophils and clinical parameters.**  **A)** Matrix of correlations between the expression of surface markers on blood eosinophils and the number of exacerbations, inhaled corticosteroid dose and total IgE from the group severe asthma. Correlations were calculated using Spearman's method. Abbreviations: R = Spearman factor, p = p-value of the test. **B-C.** Graphical representations **(B)** and confidence intervals **(C)** of the significant correlations shown in Figure 2. Statistical analyses were performed with the Spearman test, R = Spearman factor. A simple linear regression is shown in red.

**Supplementary Table 1:** Table of correlation analysis results of Eosinophil Surface Marker Expression Across Clinical Groups (Severe Asthma, CRSwNP, Healthy Subjects). Correlations were calculated using Spearman's method. Spearman correlation coefficients (R) and corresponding p-values are shown for each pair of markers. Significant p-value are highlighted in blue.
