# Supplementary material for: Associations Between Blood Eosinophil Surface Proteins and Clinical Traits in Severe Asthma and Chronic Rhinosinusitis With Nasal Polyposis
Source: Allergy. 2025 Aug 2;80(12):3454–7. doi: 10.1111/all.70001 (PMC12666750; doi:10.1111/all.70001)
Supplement: Supplementary file 1 — Data S1: all70001‐sup‐0001‐DataS1.zip. [file ALL-80-3454-s001.zip › Revised_Delaunay_et_al._2025_Supplementary_Figures.pdf]

Supplementary Figure 1 :

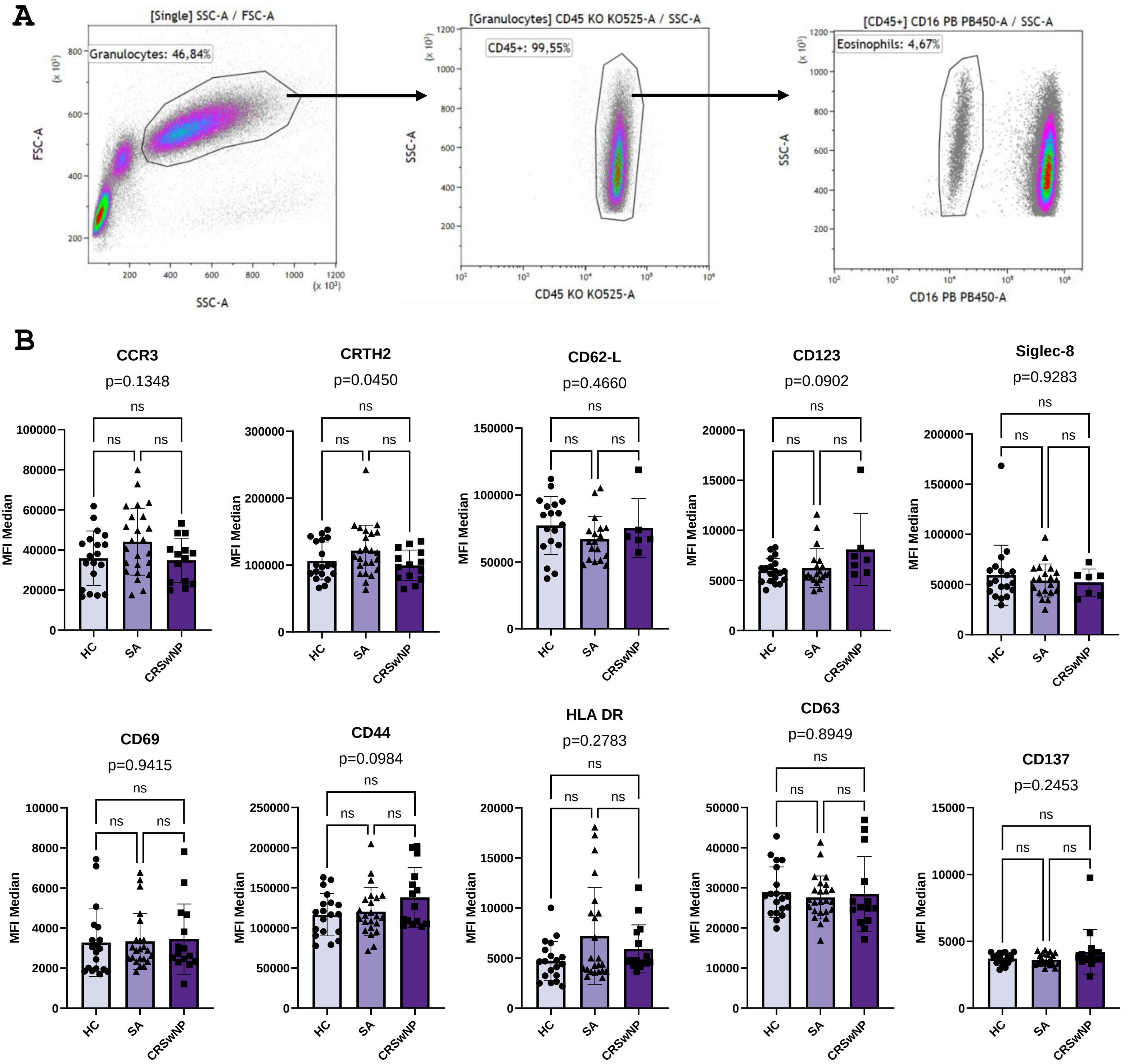

Supplementary Figure 2 :

A

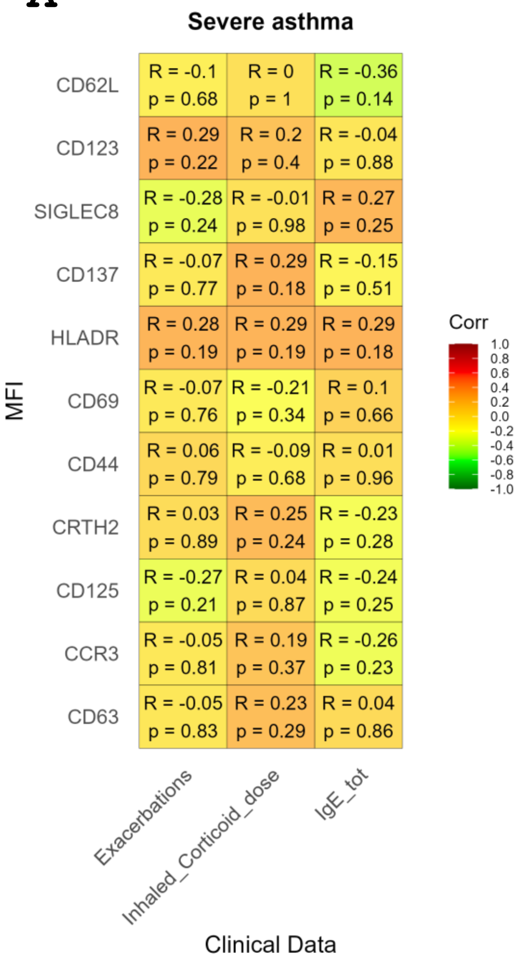

B

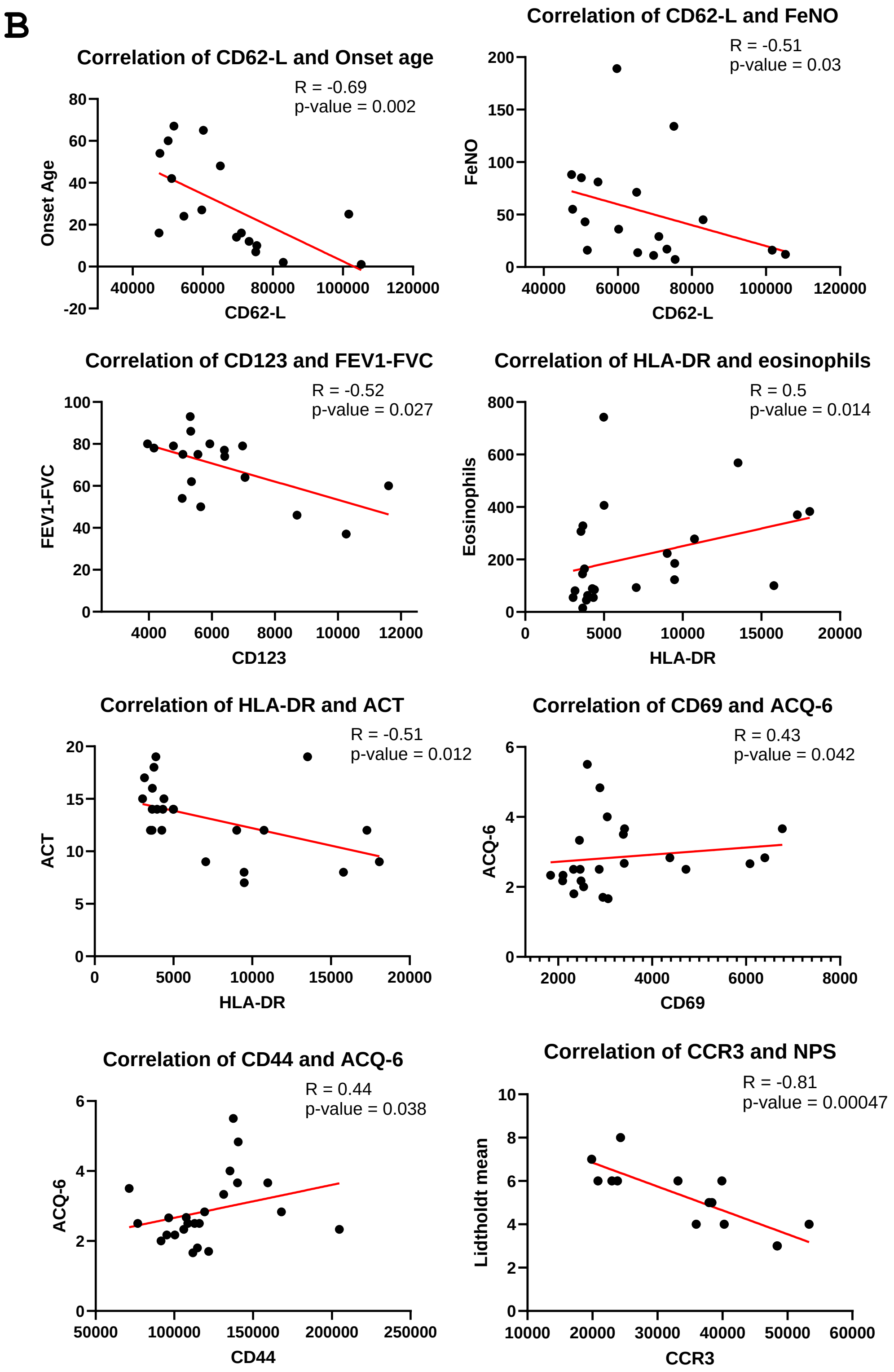

C

Group	MFI	Clinical Data	Confidence interval
Severe Asthma	CD62-L	Age onset	[-0.88 ; -0.32]
	CD62-L	FeNO	[-0.79 ; -0.06]
	CD123	FEV1/FVC	[-0.79 ; -0.07]
	HLA-DR	Eosinophils	[0.11 ; 0.76]
	HLA-DR	ACT	[-0.76 ; -0.13]
	CD69	ACQ-6	[0.02 ; 0.71]
	CD44	ACQ-6	[0.03 ; 0.72]
CRSwNP	CCR3	NPS	[-0.94 ; -0.49]
	CD63	NPS	[0.02 ; 0.83]

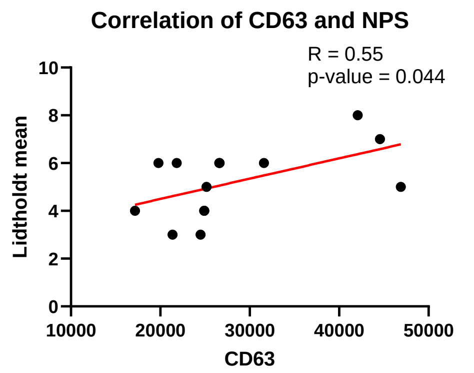

Supplementary Table 1 :

		SA		CRSwNP		HS	
Marker1	Marker2	R	p value	R	p value	R	p value
CD63	CCR3	0,14	0,50	-0,65	**0,01**	0,23	0,35
CD63	CD125	0,26	0,22	-0,13	0,67	0,32	0,18
CD63	CRTH2	0,50	**0,01**	-0,44	0,12	0,40	0,09
CD63	CD44	-0,21	0,33	-0,32	0,29	0,26	0,28
CD63	CD69	0,49	**0,02**	0,39	0,19	0,49	**0,03**
CD63	HLADR	0,34	0,11	0,32	0,29	0,19	0,43
CD63	CD137	0,73	**0,00**	0,66	**0,02**	0,12	0,61
CD63	SIGLEC8	0,26	0,27			0,32	0,18
CD63	CD123	0,13	0,58			0,42	0,08
CD63	CD62L	-0,11	0,65			0,05	0,83
CCR3	CD125	0,34	0,10	0,34	0,24	0,08	0,73
CCR3	CRTH2	0,76	**0,00**	0,64	**0,02**	0,81	**0,00**
CCR3	CD44	-0,14	0,52	0,62	**0,03**	0,06	0,80
CCR3	CD69	0,11	0,60	-0,20	0,51	-0,36	0,13
CCR3	HLADR	0,39	0,07	0,06	0,85	-0,15	0,55
CCR3	CD137	0,02	0,92	-0,42	0,15	0,15	0,55
CCR3	SIGLEC8	-0,39	0,09			-0,21	0,40
CCR3	CD123	0,23	0,33			-0,09	0,71
CCR3	CD62L	0,21	0,40			-0,24	0,34
CD125	CRTH2	0,21	0,32	0,03	0,92	0,39	0,10
CD125	CD44	-0,08	0,72	0,40	0,18	-0,28	0,25
CD125	CD69	0,28	0,20	0,18	0,57	0,56	**0,01**
CD125	HLADR	0,15	0,49	-0,40	0,18	0,34	0,15
CD125	CD137	0,27	0,21	0,15	0,62	0,19	0,43
CD125	SIGLEC8	-0,14	0,55			0,56	**0,01**
CD125	CD123	-0,13	0,58			0,15	0,53
CD125	CD62L	-0,16	0,52			0,01	0,97
CRTH2	CD44	-0,25	0,24	0,20	0,51	-0,21	0,40
CRTH2	CD69	0,27	0,20	-0,06	0,85	-0,13	0,59
CRTH2	HLADR	0,36	0,09	-0,14	0,64	-0,21	0,40
CRTH2	CD137	0,24	0,26	-0,49	0,09	0,07	0,78
CRTH2	SIGLEC8	-0,10	0,66			-0,06	0,80
CRTH2	CD123	0,26	0,28			-0,13	0,60
CRTH2	CD62L	-0,02	0,93			-0,31	0,21
CD44	CD69	0,15	0,48	0,15	0,59	-0,02	0,92
CD44	HLADR	0,22	0,32	0,08	0,78	0,10	0,68
CD44	CD137	0,12	0,59	-0,08	0,79	0,07	0,78
CD44	SIGLEC8	0,01	0,96			-0,05	0,83
CD44	CD123	0,18	0,46			0,45	0,05
CD44	CD62L	0,26	0,31			0,14	0,59
CD69	HLADR	0,23	0,28	-0,24	0,40	0,48	**0,04**
CD69	CD137	0,42	**0,04**	0,67	**0,01**	-0,01	0,97
CD69	SIGLEC8	0,35	0,14			0,83	0,00
CD69	CD123	-0,04	0,87			0,26	0,27
CD69	CD62L	-0,18	0,50			0,25	0,32
HLADR	CD137	0,32	0,14	0,10	0,73	0,41	0,08
HLADR	SIGLEC8	0,05	0,83			0,68	**0,00**
HLADR	CD123	0,75	**0,00**			0,33	0,17
HLADR	CD62L	-0,05	0,84			0,65	**0,00**
CD137	SIGLEC8	0,22	0,37			0,34	0,15
CD137	CD123	0,19	0,44			0,68	**0,00**
CD137	CD62L	-0,11	0,69			0,57	**0,01**
SIGLEC8	CD123	0,13	0,58			0,40	0,09
SIGLEC8	CD62L	-0,03	0,91			0,49	**0,04**
CD123	CD62L	0,19	0,45			0,57	**0,02**
